# Disability among Syrian refugees living in Sultanbeyli, Istanbul: Results from a population-based survey

**DOI:** 10.1371/journal.pone.0259249

**Published:** 2021-11-01

**Authors:** Sarah Polack, Nathaniel Scherer, Hisem Yonso, Selin Volkan, Isotta Pivato, Ahmad Shaikhani, Dorothy Boggs, Ammar Hasan Beck, Oluwarantimi Atijosan-Ayodele, Gülten Deniz, Ahmed Örücü, İbrahim Akıncı, Shaffa Hameed, Ceren Acarturk, Andrea Patterson

**Affiliations:** 1 International Centre for Evidence in Disability, London School of Hygiene & Tropical Medicine, London, United Kingdom; 2 Relief International, Istanbul, Turkey; 3 Maidstone and Tunbridge Wells NHS Trust, Royal Tunbridge Wells, United Kingdom; 4 Mülteciler Derneği, Sultanbeyli, Turkey; 5 Department of Psychology, Koç University, Istanbul, Turkey; Queen’s University at Kingston, CANADA

## Abstract

**Objectives:**

To estimate the prevalence of disability among Syrian refugees living in Sultanbeyli district, Istanbul and compare people with and without disabilities in terms of demographic and socio-economic characteristics.

**Methods:**

Using the municipality refugee database as the sampling frame, 80 clusters of 50 people (aged 2+ years) were selected using probability proportionate to size sampling of clusters and random selection of households within clusters. Disability assessment included: i) self-reported difficulties in functioning (using the Washington Group Short Set-Enhanced tool and Child Functioning Modules), ii) Rapid Assessment of Musculoskeletal Impairment and iii) screening for symptoms of common mental disorders for children aged 8–17.

**Results:**

The overall prevalence of disability was 24.7% (95% CI 22.1–27.4), when including people self-reporting a lot of difficulty/cannot do in at least functional domain (15%, 95% CI 13.1–17.2), moderate/severe MSI (8.7%, 95% CI 7.6–9.9), and/or symptomatic anxiety, depression and PTSD among children 8–17 (21.0%, 95% CI 18.2–23.9). Men with disabilities were significantly less likely to be in paid work compared to their peers without disabilities (aOR 0.3 95% CI 0.2–0.5). Overall 60% of households included at least one person with a disability. Households with at least one person with a disability had a significantly higher dependency ratio, lower proportion of working-age adults in paid work, and were more likely to be female headed and in receipt of social protection schemes (p<0.05).

**Conclusion:**

Disability is common among Syrian refugees in Sultanbeyli. People with disabilities in this setting experience greater vulnerability to poverty and exclusion from work, highlighting an urgent need for inclusive services, programmes and policies that are developed and implemented in partnership with people with disabilities.

## Introduction

People with disabilities include those who have long-term physical, mental, intellectual or sensory impairments which, in interaction with various barriers, may hinder their full and effective participation in society on an equal basis with others [1]. The World Health Organization estimates that 15% of the world’s population are living with a disability [2]. However, humanitarian crises, including conflict and displacement, may result in increased prevalence and severity of disability. This may occur directly, for example through war-related injuries or psychological trauma [3–5]. Further, the breakdown or loss of infrastructure, health systems, social networks, adapted environments and assistive technology, all potentially common in situations of conflict and displacement, can also cause and/or exacerbate disability [6].

The Charter on the Inclusion of Persons with Disabilities in Humanitarian Action commits that humanitarian action must be inclusive of persons with disabilities [7] and the Sustainable Development Goals commitment is to ‘leave no-one behind’ [8]. Yet, evidence shows that people with disabilities are disproportionately marginalised and excluded, experience greater vulnerability to poverty [9] and unequal access to protection, services, employment and social protection compared to people without disabilities [2]. A key barrier to planning and advocacy for disability-inclusive humanitarian response is the lack of reliable data on disability; including prevalence estimates and data disaggregated by disability status identifying inequalities and disparities in inclusion, where they exist [10].

The war in Syria has resulted in the displacement of more than 5 million Syrians. Turkey currently hosts an estimated 64% (3.6 million) of all Syrian refugees and the vast majority (>95%) live in urban settings amongst the host population [11]. An estimated 500,000 Syrian refuges are living in Istanbul [12]. Data on disability among Syrian refugees in Istanbul, or elsewhere in Turkey, are lacking. A limited number of reports present prevalence estimates of 1–5% [13, 14]. However, the methods used to derive these estimates are unclear and they may include only people officially registered as disabled (e.g. with social protection schemes) which is likely to underestimate the prevalence [15]. In contrast, a recent population survey among Syrian refugees living in Jordan and Lebanon using the Washington Group questions (an internationally recognised method to assess disability [16]), found that 22.8% had a disability [17]. The study also found that poorer households were more likely to have children with disabilities and that adults with disabilities were more likely to face barriers to work [17]. Research in Turkey by Crock et al (2015) highlighted that refugees with disabilities, who had previously worked in Syria, faced particular challenges, including stigma, in securing income generating opportunities [13].

Reliable population level data on disability among Syrian refugees living in Turkey are needed in order to inform disability inclusive policy-making, programming and advocacy in this setting. The aim of this study was to estimate the prevalence of disability and generate disability disaggregated data for key socio-economic and demographic characteristics among displaced Syrians living among the host community in Sultanbeyli District, Istanbul.

## Methods

A population-based survey among Syrian refugees living in Sultanbeyli district was conducted between September-November 2019. Sultanbeyli is a district located on the outskirts of Istanbul on the Anatolian side. At the time of the study approximately 20,000 Syrian refugees lived amongst the host community.

### Sampling

A sample size of 4,000 was required based on a conservative estimated all-age disability prevalence of 5%, a precision of 20% around the estimate, 95% confidence, 20% non-response, and a design effect of 1.7 Multi-stage cluster sampling was used to select 80 clusters of 50 people. A cluster was defined as a street within Sultanbeyli. Using the municipality refugee registration database as the sampling frame, we used probability proportionate to size sampling to select 80 streets. Within each cluster, households were randomly selected until at least 50 people were included. When a street did not include 50 people, connecting and adjacent streets were randomly selected until the target number was achieved. If any selected households were found to be no longer living in the district, replacement household(s) within the same cluster were selected.

Participants were contacted in advance by phone where possible and then visited in their households for interview. Eligible participants who were not available after two repeat visits to the household were recorded as non-responders.

### Disability assessment

Disability is multifaceted and complex to define and measure [15]. Direct report (e.g. asking people ‘do you have a disability’) may under-estimate prevalence, as people may not want to declare a disability (e.g. due to stigma) or do not consider themselves disabled. The International Classification of Functioning, Disability and Health Framework (ICF) is widely used to conceptualise disability [18]. It includes three components of disability: impairments, activities and participation and highlights that personal *(e*.*g*. *assistive technology*, *education)* and environmental factors *(e*.*g*. *accessible infrastructure*, *policies)* influence the extent to which people with impairments/health conditions experience activity and participation restrictions. Different measurement approaches capture different ICF components. For example, objective clinical assessments determine the presence, severity and type of impairment. This approach provides important information for planning health/rehabilitation services, however it doesn’t capture the impact of an impairment on a person’s activities/ participation and requires data collectors with clinical knowledge. Another approach is self-reported functioning, for example asking people about their level of difficulties with activities indifferent functional domains. This approach can be administered more quickly and without need for clinical expertise.

In this study, we collected data on self-reported functioning using the Washington Group Short Set Enhanced Set of questions (WG-SS–Enhanced; adults 18+ years) and the Washington Group/UNICEF Child Functioning Module (CFM; children 2–17 years) [16]. These are internationally recognised tools widely used in surveys, including with refugee populations [17], to collect comparable data on disability. They ask participants to self-report on difficulties in basic universal activities in the domains of: seeing, hearing, walking/climbing stairs, self-care, remembering/concentrating, communication, upper body activities, self-care and affect. The CFM additionally asks about behaviour, playing, making friends, learning and accepting change. Difficulty is rated as either: ‘none’, ‘some’, ‘a lot’ or ‘cannot do’. Participants are also asked to report on frequency and severity of depression and anxiety. We did not ask the anxiety and depression questions usually included in the CFM (for children 5–17) because children underwent more in-depth mental health assessment, as described below. Children <2 years were not included because of the lack of reliable tools to assess disability in this age group.

Based on specific need for data to inform local physical rehabilitation and mental health programmes, we also conducted more in-depth screening of common mental disorders (CMD) among children/adolescents and clinical assessment of musculoskeletal impairment (MSI).

For children aged 8–17 years, symptoms of three CMDs (anxiety, depression and post-traumatic stress disorder) were assessed using screening tools that had been previously validated for refugee populations [19–21]. For post-traumatic stress disorder (PTSD) we used the 8-item Child Revised Impact of Event Scale (CRIES). For depression we used an abbreviated 10-item version of the Center for Epidemiologic Studies Depression Scale (CES-DC) and for anxiety, an abbreviated 18-item version of the Screen for Child Anxiety Related Disorders (SCARED). These abbreviated versions have been validated for use with Syrian refugee children in Lebanon [22]. These assessments were restricted to children only, to complement a recent study on CMD among adult Syrians living in the same district [23]. This paper will report on the combined prevalence of symptoms of CMD only; full details of the study methods and findings are published separately [24].

For MSI assessment, we used the validated Rapid Assessment of Musculoskeletal Impairment tool (RAM) for participants aged 2+ years [25]. This included six screening questions about difficulty and pain using the limbs or body, use of assistive products and experiences of convulsions, collected as part of the household survey. Participants who screened positive were visited at their home the next day (or later date as required by participant) by a trained physiotherapist for a standardised assessment including physical examination and observation of activities to determine presence, severity and diagnosis of MSI [25]. This paper will report on prevalence of moderate/severe MSI only; full details of the RAM methods and findings will be published separately.

For the purposes of this study, aligning with previous research [15], disability was defined as:

- participants self-reporting significant functional limitations: including “a lot of difficulty” or “cannot do” in any Washington Group functional domain (all ages) and/or daily experiences of depression and/or anxiety, with severity described as “a lot” (adults aged 18+) [16].
- participants identified as having a moderate/severe MSI (all ages)
- children (aged 8–17) scoring at or above validated cut-off scores for symptomatic anxiety (cut-off score of 12), depression (cut-off score of 10) and PTSD (cut-off score 17) [24].

### Other variables

Socio-demographic and economic data were also collected including age, sex, education, marital status, employment, income, household indicators of relative wealth (e.g. asset ownership, heating source, building type), sex of household head (self-declared) and whether household is in receipt of social protection schemes (including Emergency Social Safety Net cash transfer scheme, food aid and socio-economic support).

### Data collection

Data collection was undertaken by 17 enumerators who underwent 10 days of training and three physiotherapists who underwent five days training to conduct the MSI assessment.

Participants aged 10 years and above were interviewed directly. For children aged under 10 years and any participant unable to communicate independently, a primary caregiver was interviewed as a proxy. The exception was the mental health screening for which all children were interviewed directly.

Data collection tools were translated into Arabic using forward and backward translation procedures and were pilot tested with members of the target population. Data were collected on android tablets using Open Data Kit software and were encrypted and uploaded to a secure cloud-based server daily.

### Data analysis

Data analysis was completed using STATA software. Disability prevalence estimates were calculated using the ‘svv’ command in STATA to account for the cluster sampling. Logistic regression analysis was used to compare individual and household demographic and socio-economic characteristics between people with and without disabilities. The comparison of individual level characteristics (e.g. work, education, marital status) by disability status were stratified by sex because of differences evidenced in other studies among Syrian populations. Socio-economic characteristics of households with and without member(s) with disabilities were also compared. A household dependency was calculated as the ration of dependents (<15 years and > 65 years) to work age adults living in the household. Principal components analysis was used to derive a socio-economic position (SEP) index from household level indicators such as asset ownership, building type and source of heating.

### Ethical considerations

Ethical approval was obtained from Istanbul Sehir University, Republic of Turkey Ministry of Interior: Directorate General of Migration Management and the London School of Hygiene & Tropical Medicine. Informed written consent was sought from participants aged 18 years and above. For participants under 18 years we sought verbal assent as well as written consent from their caregiver. Participants identified as having health service needs, including rehabilitation and mental health services, were referred to relevant local services. Participants identified as having a disability were provided with verbal and written information about the services available at a refugee association (Mülteciler Derneği) based in Sultanbeyli and advised to visit to discuss their needs. Services provided by Mülteciler Derneği include mental health and psychosocial support, physical rehabilitation, primary health care, legal advice and vocational training as well as referral to other health services. People identified through the survey to have urgent mental health, physical rehabilitation or safeguarding needs were referred to the specific relevant services at Mülteciler Derneği and assigned a caseworker.

## Results

### Study population

In total, 4018 people were enumerated out of which 3084 were included in the survey (response rate 77%), 613 (165%) were unavailable and 321 (8%) refused to participate. Compared to those who took part in the survey, non-participants were, on average, significantly older (21.8 years, 95%CI 21.3–22.4 versus 25.4 years, 95%CI 24.4–26.4, p<0.001) and more likely to be male (47% vs 65%, p<0.001). Overall, however, the sample age and sex distribution was broadly comparable to the registration database used as the sampling frame (Table 1).

**Table 1:** Comparison of the age and sex distribution of the study sample and sampling frame.

	Total				Males				Females			
	Registration database		Study sample		Registration database		Study sample		Registration database		Study sample	
Age (years)	N	%	N	%	N	%	N	%	N	%	N	%
2–9	4,793	26%	875	28%	2,497	26%	442	31%	2,296	26%	433	26%
10–19	4,440	24%	773	25%	2,316	24%	372	26%	2,124	24%	401	24%
20–29	3,558	19%	507	16%	1,735	18%	198	14%	1,823	20%	309	19%
30–39	2,844	15%	446	14%	1,574	16%	207	14%	1,270	14%	239	15%
40–49	1,545	8%	239	8%	795	8%	107	7%	750	8%	132	8%
50–59	935	5%	161	5%	484	5%	78	5%	451	5%	83	5%
60+	547	3%	81	3%	267	3%	38	3%	280	3%	43	3%
Total	18,662	100%	3084	100%	9,668	48%	1443	47%	8,994	52%	1640	53%

Half the study population were under 17 years and only 3% were above 60 years and 53% of the study population were female (Table 1). The average number of years since leaving Syria was 4.1 (95% CI 4.0–4.2).

### Prevalence of disability

Table 2 shows the prevalence estimates for disability overall and separately by self-reported functional limitations, MSI and CMD (among children). The overall prevalence of disability according to the study definition, (reporting a lot of difficulty/cannot do in any WG-SS Enhanced/CFM domain, moderate/severe MSI, and/or symptomatic anxiety, depression and PTSD among children 8–17) was estimated to be 24.7% (95% CI 22.1–27.4). Prevalence increased significantly with age and was 50.8% (95% CI 44.7–56.8) among adults aged 50 years and above.

**Table 2:** Prevalence of disability among Syrian refugees living in Sultanbeyli, Istanbul.

	2–17 years		18–49 years		50+ years		All ages	
	N	% (95% CI)	N	% (95% CI)	N	% (95% CI)	N	% (95% CI)
Self-reported functional limitation^a^								
Male (n = 1437)	59	7.7% (5.8–10.2)	92	16.4% (12.9–20.7)	39	33.9% (25.3–43.6)	190	13.2% (11.1–15.6)
Female (n = 1639)	66	8.6% (6.4–11.4)	150	20.1% (16.4–24.3)	56	44.4% (36.5–52.6)	272	16.6% (14.2–19.4)
All (n = 3077)^b^	125	8.2% (6.5–10.2)	242	18.5% (15.4–22.1)	95	39.4% (33.4–46.2)	462	15.0% (13.1–17.2)
Moderate/severe Musculoskeletal Impairment								
Male (n = 1410)	17	2.2 (1.3–3.8)	81	14.9 (11.8–18.3)	30	26.5 (19.2–35.4)	128	9.1 (8.9–9.2)
Female (n = 1611)	32	4.2% (2.9–6.0)	59	8.1% (6.1–10.9)	43	34.9% (27.1–43.7)	134	8.3% (6.7–10.2)
All (n = 3022)	49	3.2% (2.4–4.3)	140	11.5% (9.3–13.1)	73	30.9% (25.4–37.0)	262	8.7% (7.6–9.9)
Symptomatic anxiety/anxiety/PTSD (children 8–17 only)								
Male (n = 412)	107	19.1% (15.1–24.0)						
Female (n = 439)	147	28.0% (22.9–33.7)						
All (n = 852)	202	23.7% (19.9–27.2)						
All disability^c^								
Male (n = 1442)	139	18.2% (15.1–21.8)	136	24.2% (20.1–28.7)	52	44.8% (35.9–54.1)	327	22.7% (20.0–25.6)
Female (n = 1640)	182	23.7% (20.4–27.4)	181	24.2% (20.1–28.8)	71	56.3% (49.2–63.2)	434	26.5% (23.3–29.9)
All (n = 3084)	321	21.0% (18.2–23.9)	317	24.2% (20.7–27.9)	123	50.8% (44.7–56.8)	761	24.7% (22.1–27.4)

^a^Reporting a lot of difficulty/cannot do in any functional domain of the WGES/CFM and/or daily experiences of depression and/or anxiety, with severity described as “a lot”;

^b^Data were missing for 7 people

^c^Defined in this study as reporting a lot of difficulty/cannot do in any WGES/CFM functional domain, moderate/severe MSI, and/or symptomatic anxiety, depression and PTSD among children 8–17; One participant did not report their gender.

The high overall disability prevalence among children was driven particularly by the high prevalence of symptomatic CMDs (21.0%, 95% CI 18.2–23.9). These were assessed through separate screening tools, which were not used for adults. If these CMD data are not included, the prevalence of disability among children (based on the WG/CFM and MSI assessment only) was 10.2% (95% CI 8.4–12.4) and the overall all-age estimate was 19.4% (95% CI 17.2–21.6).

### Self-reported functional limitations

**Fig 1:**
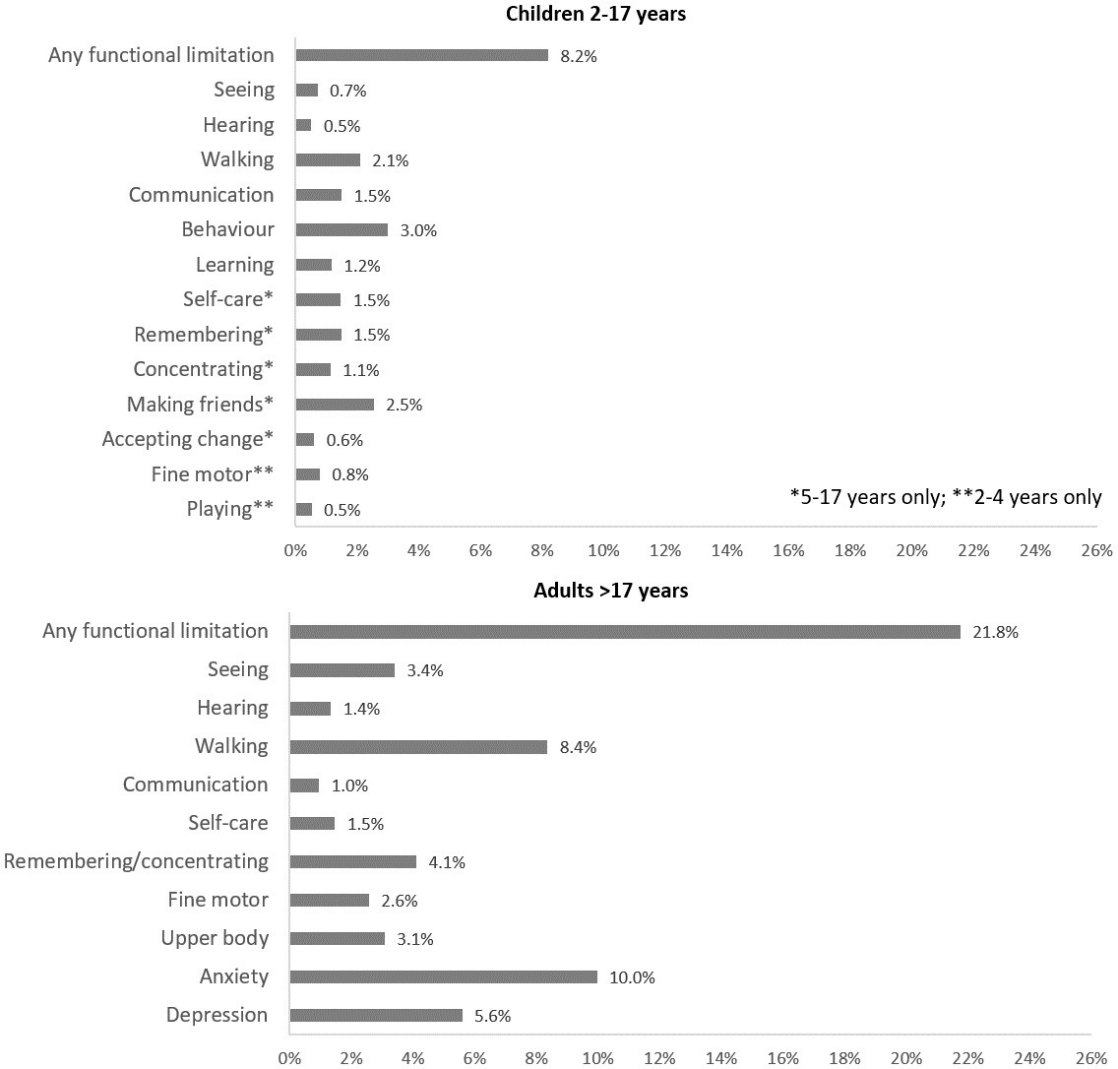
Proportion of participants reporting a lot of difficulty or cannot do by functional domain.

The overall estimated prevalence of self-reported functional limitations (a lot of difficulty/cannot do in at least one WG-SS Enhanced/CFM domain) was 15.0% (95% CI 13.1–17.2) (Table 2); among children/adolescents (2–17 years) this was 8.2% (95% CI 6.5–10.2) and among adults (18+ years) this was 21.8% (95% CI 18.8–25.0) (Table 2). The most commonly reported functional limitations among children/adolescents were behaviour (3.0%), making friends (2.5%) and walking (2.1%). Amongst adults (18+) difficulties were most commonly reported in the domains of anxiety (10.6%), walking/climbing (9.2%) and depression (6.3%) (Fig 1).

### Comparison of people with and without disabilities

Overall, adults with disabilities were significantly less likely to be in paid work (Odds Ratio, OR 0.6, 95% CI 0.4–0.8) (Table 3). Upon stratification by sex however, this trend was only evident among men; 39% of men with disabilities were in paid work compared to 71% among their peers without disabilities (OR 0.3, 95% CI 0.2–0.5). Among women, only 5% of people with and without disabilities were in paid work.

**Table 3:** Individual level characteristics (education, marital status and work) among adults (>17 years) with and without disability disaggregated by sex.

	All participants:					Men only					Women only				
	People without disabilities		People with disabilities		Age and sex adjusted Odds Ratios (95% CI)	Men without disabilities		Men with disabilities		Age adjusted Odds Ratios (95% CI)	Women without disabilities		Women with disabilities		Age adjusted Odds Ratios (95% CI)
	N =		N =			N =		N =			N =		N =		
	N	(%)	N	%		N	%	N	%		N	%	N	%	
**Paid work**															
Not currently in paid work	734	66%	350	81%	Reference	143	29%	111	61%	Reference	591	95%	239	95%	Reference
Currently in paid work	375	(34%)	84	19%	0.6 (0.4–0.8)^a^	347	71%	72	39%	0.3 (0.2–0,5)^a^	28	5%	12	5%	1.1 (0.6–2.1)
**Highest education level**															
Never attended	89	8%	59	14%	1.1 (0.7–1.8)	34	7%	15	8%	1.0 (0.5–2.0)	55	9%	44	18%	1.1 (0.7–1.9)
Primary	495	45%	200	46%	Reference	224	46%	81	44%	Reference	271	44%	119	47%	Reference
Middle/Secondary	453	41%	152	35%	0.9 (0.7–1.2)	194	40%	78	42%	1.2 (0.8–1.7)	259	42%	74	29%	0.8 (0.6–1.1)
Post-Secondary	72	7%	24	3%	0.9 (0.5–1.5)	38	8%	10	5%	0.7 (0.3–1.7)	34	5%	14	6%	1.1 (0.6–2.3)
**Marital status**															
Married/living together	912	82%	340	78%	Reference	387	79%	153	84%	Reference	525	85%	187	75%	Reference
Divorced/separated	21	2%	17	4%	2.0 (1.0–4.2)	2	0%	0	0%	N/A	19	3%	17	7%	2.1 (1.0–4.5)
Widowed	41	4%	38	9%	1.2 (0.8–2.1)	2	0%	3	2%	N/A	39	6%	35	14%	1.2 (0.7–2.0)
Single	135	12%	39	9%	1.5 (1.0–2.4)	99	20%	27	15%	1.7 (0.8–3.4)	36	6%	12	5%	1.3 (0.6–2.9)

^a^ P<0.05.

Adults with disabilities were more likely than peers without disabilities to be divorced/separated, although this difference was only of borderline significance (OR 2.0, 95% CI 1.0–4.2). This trend was only evident among women (OR 2.1, 95% CI 1.0–4.5), and marital status did not differ between men with and without disabilities. No association was observed between disability status and the highest level of education received among men or women. We also assessed the association between paid work and level education (adjusted for disability, age and sex) and did not find a statistically significant association (data not shown).

In terms of household level variables (Table 4), people with disabilities were more likely to live in households that were in receipt of socio-economic support (OR 2.3, 95% CI 1.4–3.7) and food aid (OR 1.5, 95% CI 1.2–1.9). No association was observed with household socio-economic position, accommodation type or monthly rent.

**Table 4:** Household level factors among people with and without disabilities.

	People without disabilities (n = 2323)		People with disabilities (n = 761)		Age and sex adjusted Odds Ratios (95% CI)
	N	%	N	%	
**Socio-economic position**					
1^st^ quartile (poorest)	625	27%	197	26%	Reference
2^nd^	603	26%	192	26%	0.9 (0.7–1.3)
3^rd^	563	24%	200	27%	1.1 (0.8–1.4)
4^th^ (least poor)	524	23%	161	21%	0.9 (0.8–1.3)
**Accommodation type**					
Flat/apartment	1837	79%	618	82%	Reference
House	400	17%	111	15%	0.9 (0.7–1.1)
Basement	62	3%	18	2%	1.0 (0.5–2.2)
Store/warehouse	16	1%	5	1%	0.7 (0.3–1.9)
**Rent (lira/month)**					
0–400	85	4%	27	4%	Reference
401–800	2003	87%	649	86%	1.2 (0.7–2.1)
>800	227	10%	76	10%	1.1 (0.6–2.3)
**Social support schemes**					
Receiving ESSN cash assistance	1425	62%	460	61%	1.0 (0.8–1.3)
Receiving Socio-economic support	79	3%	56	7%	2.3 (1.4–3.7) ^c^
Receiving Food aid	634	27%	289	38%	1.5 (1.2–1.9) ^c^

Data were missing for 7 people with disabilities and 6 people without disabilities;

^b^ESSN: Emergency Social Safety Net cash transfer scheme

^c^p<0.05.

### Comparison of households with/without a member with a disability

In total 413 (60%) households included at least one person with a disability. Households with at least one member with a disability had, on average, significantly more household members, a higher number of total dependents and a higher dependency ratio, compared to households without members with disability (p<0.01) (Table 5). They also had a lower number of adults (of working age) currently in paid work. A higher proportion of households including a person with a disability member were female-headed compared to those without (p = 0.02). In terms of social protection, households including at least one person with a disability were significantly more likely to be in receipt of Emergency Social Safety Net cash transfer, socio-economic support schemes and food aid (p<0.01).

**Table 5:** Comparison of characteristics of households with and without a member with a disability.

	Households without members with a disability (n = 274)	Households with members with a disability (n = 413)	
	Mean (95% CI)	Mean (95% CI)	p-value^c^
Household size	4.7 (4.5–4.9)	5.6 (5.4–5.8)	<0.001
Proportion female	48.8% (46.6–51.0)	49.9% (48.0–51.8)	0.46
No. dependents	2.0 (1.8–2.2)	2.5 (2.3–2.6)	<0.001
Dependency ratio^a^	0.9 (0.8–1.0)	1.0 (0.9–1.1)	0.04
SES index score	0.01 (-0.21–0.23)	-0.01 (-0.18–0.16)	0.88
Average household rent	687.3 (610.9–763.6)	648.3 (633.4–663.2)	0.24
Proportion working among working age	**Median (SD)**	**Median (SD)**	**p-value** ^d^
	0.4 (0.23)	0.3 (0.26)	<0.001
Female headed household	**N (%)**	**N (%)**	**p-value** ^e^
	42 (15%)	94 (23%)	0.02
Social protection schemes			
Receiving ESSN^b^ cash assistance	118 (43%)	242 (59%)	<0.001
Receiving socio-economic support	4 (2%)	26 (6%)	0.002
Receiving food aid	57 (21%)	150 (36%)	<0.001

^a^ Dependency ratio: ratio of dependents (<15 years and >65 years) to working age adults living in the household;

^b^ESSN: Emergency Social Safety Net cash transfer scheme;

^c^ p-value from student t-test,

^d^ p-value from Mann-Whitney test for skewed data,

^e^ p-value using chi2 test; SD: Standard deviation.

## Discussion

Disability was common among Syrian refugees living in Sultanbeyli district at 24.7%; including people self-reporting severe functional limitations (15%), identified as having a moderate/severe MSI (8.6%) and/or symptomatic CMDs among children (21%). Prevalence increased significantly with age. Men with disabilities were less likely to be in paid work. Households with members with at least one disabled person were on average larger, had a higher dependency ratio and were more likely to have a female head of household. They were also more likely to be in receipt of social protection support/schemes.

The high prevalence of disability in our study aligns with other recent data from Syrian populations. Surveys in Lebanon and Jordan in 2017 found an average prevalence of 22.9% among Syrian refugees [17]. These surveys used the WG-SS Enhanced and CFM, as applied in the current study. However, they also included a question on fatigue, and found significant difficulty with fatigue was commonly reported by adults (11%). This may partly explain the higher prevalence when compared to our study’s estimated prevalence of self-reported functional limitations (15%). A national survey within Syria in 2019 estimated a disability prevalence of 27% among people aged 12+ years using the WG short set of questions only (which do not include the questions on upper body, anxiety or depression) [26]. The higher prevalence within Syria, despite the lower number of functional domains assessed, may reflect the younger age of our study population (which included children 2–12 years), increased exposure to the conflict, and/or that people with disabilities may be more likely to remain behind.

For adults, the prevalence estimates for anxiety (10.6%) and depression (6.3%) were similar to those from Jordon and Lebanon (11.4% and 5.9% respectively). Anxiety and depression were among the most commonly reported of the WG functional domains in both studies, highlighting the need for quality Mental Health and Psychosocial Support (MHPSS) for displaced Syrians. However, our estimates are considerably lower than those of a 2018 survey conducted among adult Syrians in Sultanbeyli, which used specific mental health screening tools (Hopkins Symptoms Checklist) for anxiety (36.1%) and depression (34.7%) [23]. This suggests that the WG questions, using the recommended cut-off, may under-estimate prevalence of symptomatic anxiety and depression. It also raises concerns about the use of the WG-Short Set of questions in crises affected populations, despite their relative brevity and operational feasibility, because of the complete absence of questions on anxiety and depression. Accordingly, the use of the WG-SS Enhanced set is encouraged in a recent initiative promoting disability data collection in humanitarian action [27].

Our estimates for symptoms of common mental disorders among children (discussed in detail in separate publication [24]) are not directly comparable to the disability surveys in Jordan and Lebanon because of the different assessment methods. However, they do fall within the range of other studies using similar mental health tools with this population [5].

Overall, these recent disability prevalence estimates for Syrian populations are generally higher than for non-refugee/conflict affected populations in low- and middle-income countries (LMIC), particularly among working age adults. For example, in a national survey in Guatemala, the prevalence of functional limitations (using the same WG tools) was 7.4% [28] compared to 15% in this study. The all-age prevalence of moderate/severe MSI in our study was 8.6% (11.5% among adults aged 18–49) compared to 3.5% found in both Cameroon and India (1.5% and 2.9% among adults 18–49 respectively) using the same Rapid Assessment of Musculoskeletal Impairment methodology [29, 30]. These differences may be due to direct conflict related injury or psychological trauma, as well as the loss of social structures, health infrastructure and/or inclusive environments that displaced populations may experience. That more than half of households in our survey included a member with a disability emphasises the critical importance of disability-inclusive programmes and services in this setting that are built on an understanding of the diverse needs and capabilities among people with disabilities.

Syrian refugees have the right to apply for a work permit in Turkey, although Bellamy et al (2017) highlight the complexities and challenges refugees face in securing paid work [31]. In our study, men with disabilities were much less likely to be engaged in paid work compared to their peers without disabilities. Fewer than 5% of women were working. These findings align with other studies that suggest both disability and gender specific barriers are experienced by Syrian refugees in accessing the labour market [13, 17]. Considering the high prevalence of disability among working aged men, this low participation in paid work is likely to have a substantial financial impact on many families, especially important when considering the additional costs associated with living with a disability (such as health and social care costs). Further, the opportunity to work can be central for rebuilding lives of refugees, increasing self-reliance and dignity [32]. Previous research highlights a tendency for disability-related humanitarian response to focus predominantly on health/rehabilitation needs with less attention given to social inclusion and livelihoods [33]. Our findings emphasise the need for refugee sensitive livelihood and economic inclusion policy and programmes that are disability inclusive. These must be developed in partnership with people with disabilities (including representation from men, women, different ages and impairment types) to identify and address barriers, needs and capacities, and recognise the diversity and complexity of disability and context.

Although customarily Syrian males more commonly assume the role of ‘head of household’, the conflict has resulted in increased numbers of female-headed households [34]. Adding to this, our study found that female-headed households were more common among households with a member with a disability. The exact reasons for this are unclear, though research in other settings has found a high risk of fathers leaving when a child with a disability is born [35]. Our finding deserves further attention, particularly considering the low proportion of women in paid work and previous research showing that, among Syrian refugees, female headed households are more vulnerable to poverty on a range of indicators (e.g. food insecurity and living below the poverty line) [34].

Poverty and disability are known to be cyclically linked [9]. However, this study found no association between disability and household socio-economic position. A possible explanation is that the asset based measure we used is more reflective of the long-term household economic situation and may not be appropriate or sensitive enough for a recently displaced population. Indeed, other findings in our study which are reflective of the multi-dimensional nature of poverty (e.g. households with disabilities had lower proportion of working-age people in paid work, were more likely to be female headed and have a higher number of dependents) suggest that people/households with disabilities are at increased risk of vulnerability to poverty.

Households with members with a disability were more likely to be in receipt of social protection schemes, which is encouraging and suggests some appropriate targeting of these schemes. However, overall coverage was still relatively low, particularly for food aid and socio-economic support schemes and this deserves further attention.

Turkey hosts the majority of Syrian refugees, with most living in urban areas among host communities [11]. Our findings have implications for policy and programmes for this population. Considering the high numbers of people and households affected by disability, disability in this population cannot be ignored; it is a human rights issue that must be considered in policies and programmes from inception through to implementation and evaluation and including financing. Routine collection of disability data, alongside age and sex, within services and programmes (e.g. health, education, social protection) is key so that disability inclusion can be monitored and advocated. Research, in other settings, suggests this has also improved targeting of vulnerable households [27]. Using the WG tools would enable consistency and comparability with other humanitarian initiatives [27]. Considering the long-term nature of the Syrian crises, the importance of including refugees in the economy, e.g. through sustainable income generation, is increasingly recognised [36]. However, our findings suggest people with disabilities are being left behind in these efforts. Accordingly, several documents detailing livelihood initiatives and experiences for refugees in Turkey, make little or no mention of disability [31, 36]. Structural changes that remove barriers to sustainable livelihoods, with sensitivity to context, culture and gender dynamics, make good sense in terms of wider economic development as well as resilience, dignity and independence. There are several ‘how to’ guidelines for this, including specifically for urban humanitarian response [37]. However, there is a major gap in the evidence-base on disability inclusive interventions in humanitarian response, that must be addressed [38]. Following the principal of “nothing about us without us”, refugees with disabilities and their organisations must be included in the development and implementation of policies and programmes that relate to them.

### Strengths and limitations

This survey contributes to the data on disability among Syrian refugees. We used standardised sampling methods and internationally recognised tools for assessing functional limitations, CMDs (children) and MSI.

There were limitations. The response rate was just below 80%, despite considerable effort to revisit households on days/times when they were most likely to be at home. If people with disabilities were more likely to be at home, this may have led to an over-estimation of disability prevalence. However, the age and sex distribution were similar to the sampling frame. The sample was selected from the municipality registration database which includes registered refugees only. However, all members of study households were included regardless of registration status. The proportion of the sample aged over 50 was small, aligning with other data on Syrian populations [17, 26], therefore some caution in the interpretation of prevalence estimates for this age group is warranted. We did not ask the CFM questions on anxiety and depression for children, because we conducted more detailed assessment using specific screening tools in these domains. This limits direct comparability of the overall prevalence estimate to other studies using the CFM and of the anxiety/depression estimates between adults and children in this study. We conducted detailed assessment of child mental health and musculoskeletal impairment, to inform specific programmes. However, impairment assessments for other domains (e.g. vision, hearing, communication) were not included due to time and resource constraints; ideally these should be collected in future where resources allow to inform service provision. Finally, it is possible that the presence of anxiety and depression may negatively impact on individuals’ perception of their physical and sensory functioning [39] which could may have led to some over-reporting of difficulties in these domains.

## Conclusion

The finding that disability is common among Syrian refugees in Sultanbeyli and that refugees with disabilities in this setting experience greater vulnerability to poverty and exclusion from work highlights an urgent need for inclusive services, programmes and policies that are developed in partnership with people with disabilities and with consideration to gender equality.
